# Demand for malaria rapid diagnostic test, health care-seeking behaviour, and drug use among rural community members with fever or malaria-like illness in Ebonyi state, Nigeria: a cross-sectional household survey

**DOI:** 10.1186/s12913-021-06865-8

**Published:** 2021-08-21

**Authors:** Ugwu I. Omale, Onyinyechukwu U. Oka, Ifeyinwa M. Okeke, Benedict N. Azuogu, Chihurumnanya Alo, Ugochukwu C. Madubueze, Irene I. Eze, Kingsley C. Okeke, Rowland Utulu, Uduak E. Akpan, Chijioke V. Iloke, Anthonia O. Nnubia, Desi O. Ibemesi, Chukwuka R. Nnabu, Ogechukwu C. Anene

**Affiliations:** 1 Department of Community Medicine, Alex Ekwueme Federal University Teaching Hospital Abakaliki (AEFUTHA), Abakaliki, Ebonyi State Nigeria; 2 Department of Community Medicine, Ebonyi State University (EBSU), Abakaliki, Ebonyi State Nigeria; 3 Department of Community Medicine, Alex-Ekwueme Federal University, Ndufu-Alike Ikwo (AE-FUNAI), Ikwo, Ebonyi State Nigeria; 4 Nigeria Field Epidemiology and Laboratory Training Programme (NFELTP), 50 Haile Selassie Street, Asokoro, Abuja, Nigeria; 5 Government House Clinic, Abakaliki, Ebonyi State Nigeria

**Keywords:** Demand, MRDT, Health care-seeking behaviour, Drug use, Fever/malaria-like illness, Nigeria

## Abstract

**Background:**

A good understanding of the demand for malaria rapid diagnostic test (MRDT), malaria health care-seeking behavior, and drug use among community members is crucial to malaria control efforts. The aim of this study was to assess the demand (use and/or request) for MRDT, health care-seeking behavior, and drug use, as well as associated factors, among rural community members (both children and adults) with fever or malaria-like illness in Ebonyi state, Nigeria.

**Methods:**

A cross-sectional household survey was conducted between October 1st and November 7th, 2018, in 18 rural geographical clusters. Data was collected using a structured interviewer-administered questionnaire. Descriptive analysis was done using summary statistics. Associated factors (socio-demographic, knowledge and opinion level) were assessed using bivariate and multivariate binomial logistic regressions while the overall effects of these factors were assessed using the “postestimation test” command in Stata.

**Results:**

A total of 1310 children under 5 years of age and 2329 children ages 5 years and above and adults (excluding pregnant women) (3639 overall) participated in the study. Among the 1310 children under 5 years of age: 521 (39.8%) received MRDT of which the caregivers of 82 (15.7%) requested for the MRDT; 931 (71.1%) sought care with public/private sector providers (excluding traditional practitioners/drug hawkers) the same/next day; 495 (37.8%) sought care at government primary health centres, 744 (56.8%) sought care with the patent medicine vendors (PMVs); 136 (10.4%) sought care with traditional practitioners; 1020 (77.9%) took ACTs (=88.2%, 1020/1156 of those who took anti-malarial drugs). Generally, lower values were respectively recorded among the 2329 children ages 5 years and above and adults (excluding pregnant women). The most important overarching predictor of the demand for MRDT and care-seeking behaviour was the knowledge and opinion level of respondent female heads of households about malaria and malaria diagnosis.

**Conclusions:**

Among the rural community members with fever or malaria-like illness in Ebonyi state, Nigeria, while majority did not receive MRDT or diagnostic testing, and sought care with the PMVs, most took anti-malaria drugs, and mostly ACTs. Interventions are needed to improve the knowledge and opinion of the female heads of households about malaria and malaria diagnosis.

**Supplementary Information:**

The online version contains supplementary material available at 10.1186/s12913-021-06865-8.

## Background

Malaria is a preventable and treatable disease but it continues to cause significant morbidity and mortality especially in the high burden countries of the World Health Organization (WHO) Africa Region (including Nigeria) [1–3]. Malaria is an endemic disease of public health importance in Nigeria and about 97% of the population is at risk of transmission which occurs year round but with peaks during the rainy seasons [4, 5].

Malaria was traditionally diagnosed presumptively (based on clinical features) or by light microscopy (of blood smear). But due to several limitations of presumptive diagnosis and malaria microscopy, malaria rapid diagnostic test (MRDT) was introduced in the early 1990s [6, 7]. Compared to microscopy, MRDT requires less skills and training, simpler to perform and interpret, requires no electricity or special laboratory equipments [microscope etc] and reagents and can be deployed in hard-to-reach rural areas [3, 6, 8]. The WHO recommendation for universal parasitological testing was informed by some fundamental changes in the global malaria epidemiology such as the declining incidence of malaria in the malaria endemic countries, the emergence of parasite resistance to artemisinin-based combination therapies (ACTs), and the increased availability of MRDT [6, 9]. The use of MRDT for the diagnosis of malaria is an invaluable part of the strategy for universal parasitological testing recommended by the WHO [6, 8, 10, 11].

Two of the objectives of the 2014–2020 Nigerian national malaria strategic plan were to test all care seeking suspected malaria patients with MRDT or microscopy by 2020 and to treat all individuals with confirmed malaria (in both public and private sectors) with effective anti-malarial drugs [4]. One of the strategic actions is to create demand for the utilization of parasitological diagnostic testing via actions targeted at both the health workers and the general public [4]. Nigeria, like other countries across the world, has been scaling up the availability of MRDT especially in the public sector. But in Nigeria the majority of patients with fever seek care in the private sector particularly with the patent medicine vendors (PMVs) [5, 12] where they are more likely to receive over-diagnosis and over-treatment for malaria with ACTs usually based on presumptive diagnosis [13, 14]. This means patients suspected of having malaria are more likely to receive parasitological diagnosis (with MRDT) and appropriate treatment (with ACTs) if they seek care with providers in the public sector (especially where these services are readily available). Therefore, promptly seeking care (within 24–48 h of onset of febrile illness), especially with public sector providers, enhances early and accurate diagnosis of malaria and prompt targeted treatment with effective anti-malarial drugs such as ACTs. This will help reduce the incidence of severe malaria, avoid preventable deaths from malaria, and reduce malaria transmission among all population groups (both children and adults) in the communities [1–3]. It will also help reduce over-diagnosis and over-treatment of malaria with ACTs and the attendant risk of drug resistance [3, 8]. It is thus invaluable to malaria control and elimination efforts.

A good understanding of the demand for MRDT as well as malaria health care-seeking behaviour and drug use (and associated factors) among community members (both children and adults), with febrile or malaria-like illness, will inform the design of tailored interventions to improve the demand for MRDT, health care-seeking behavior and targeted treatment with effective anti-malarial drugs [2]. However, there is limited information about the demand (use and/or request) for MRDT, health care-seeking behaviour, and drug use among community members especially in rural settings/communities where MRDT services are readily available in Ebonyi state as well as in Nigeria as a whole. This is particularly so with regards to children above 5 years old and adults as most related studies across the world only involved children under 5 years of age. Also, national surveys [5, 12] only assessed the use of MRDT, care-seeking behaviour and drug use among children aged less than 5 years in villages/settlements irrespective of the availability of MRDT services. Moreover, the findings among the children less than 5 years of age might show marked variations from those of the older children and adults in the communities and hence are not truly representative. The extent and direction of such variation, if any, is elucidated in this report. Also, in the context of the recommendation for universal diagnostic testing and the objectives of the 2014–2020 national malaria strategic plan to test all care seeking patients suspected of having malaria by 2020, it is necessary to assess the demand for MRDT among both adults and children.

The aim of this study was to assess the demand (use and/or request) for MRDT, health care-seeking behaviour, and drug use, as well as their determinants, among rural community members (adults and children) with fever or malaria-like illness, in communities where MRDT services were available, in Ebonyi state, Nigeria.

## Methods

### Study design

This study was an analytical cross-sectional study within a cluster randomized controlled trial which has been described elsewhere [15]. It was conducted at baseline, between October 1st and November 7th, 2018, in Ebonyi state, Nigeria, when the trial was being implemented. The purpose of the trial was to sensitize/educate social groups (social group intervention) and train health care providers in health communication about MRDT with clients (social group/provider intervention) and evaluate whether these interventions would increase the demand (use and/or request) for MRDT among community members with fever/malaria-like illness in Ebonyi state, Nigeria.

This article describes the demand (use and/or request) for MRDT, health care-seeking behaviour and drug use among rural community members with fever/malaria-like illness in Ebonyi state, Nigeria.

### Study procedure

The trial involved 18 rural geographical clusters which were randomly selected from 34 eligible clusters (villages/groups of villages) (with at least 250 households or a population of 1500 people) serving as the proximate catchment area for at least one public primary health facility and one PMV offering MRDT services in Ebonyi state, Nigeria. In Ebonyi state rainy season occurs between April and October and dry season between November and March. Malaria is endemic in the state with year round transmission which peaks during the rainy seasons. Before the delivery of trial interventions, a population-based household survey was conducted, using a structured interviewer-administered questionnaire, to assess community members’ demand for MRDT, health care-seeking behaviour and drug use.

### Study participants and sample size

In each of the selected clusters, all the households were visited and the study included those that reported any case of fever or malaria-like illness among children and adults (excluding pregnant women) in the 2 weeks preceding the population-based household survey. All the household members with reported fever/malaria-like illness, in the 2 weeks preceding the survey, participated in the study. For each household, informed consent was obtained from the female head (the mother of the house or the female primary caregiver aged 15 years and above) who was the respondent to the survey. Data was collected about fever or malaria-like illness management including health care-seeking and demand (use and/or request) for MRDT; basic socio-demographic characteristics; and respondent’s knowledge and opinion about malaria and malaria diagnosis (respectively assessed through their responses to a set of questions and statements).

In the parent study (the trial), sample size estimation and outcome assessment were done separately for two population groups: children under 5 years of age, and children ages 5 years and above and adults (excluding pregnant women). A total of 1310 children under 5 years of age and 2329 children ages 5 years and above and adults (excluding pregnant women) (an overall total of 3639) who participated in the survey were included in the analysis of this report.

### Statistical analysis

The data was double-entered using Microsoft Excel 2007 (Microsoft Inc., Redmond, WA, USA) and, verified and analysed using Stata/SE version 15.1 (Stata Corp, College Station, TX, USA). Descriptive analysis was done using frequencies and proportions/percentages. Bivariate and multivariate binomial logistic regressions (controlling for the other factors such as age group, sex, educational level, occupation, and knowledge/opinion level (knowledge and opinion score of < 50% of the total of 47 was poor knowledge, 50–< 70% was fair, > = 70% was good)) were done to assess the factors that were associated with the demand for MRDT and with health care-seeking behaviour at 5% level of significance. The “postestimation test” command in Stata was used to assess the overall effects of the independent factors. Crude and adjusted odds ratios, 95% confidence intervals and *p*-values are reported.

## Results

### Socio-demographic or background characteristics

An overall total of 3639 community members participated in the study, children under-5 years of age were 1310 and children ages 5 years and above and adults (excluding pregnant women) were 2329. The results are presented separately for these two populations. Their socio-demographic or background characteristics are presented in Table 1. Among the 1310 children under 5 years of age, the majority of them, 358 (27.3%), were aged 48–59 months followed by 313 (23.9%) who were aged 36–47 months. The majority of them, 696 (53.1%), were males. The majority of the respondent female heads of households, 542 (41.4%), had secondary education followed by 494 (37.7%) who had primary education. Similarly, 552 (42.1%) were farmers followed by 498 (38.0%) who were traders. Also, 498 (38.0%) had good knowledge and opinion (knowledge and opinion score of > = 70%) about malaria and malaria diagnosis, 695 (53.1%) had fair knowledge and opinion (score of 50–< 70%), and 117 (8.9%) had poor knowledge and opinion (score of < 50%).

**Table 1:** Socio-demographic/background characteristics of participants^a^, overall *N* = 3639

Children under 5 years of age, *N* = 1310			Children ages 5 years & above & adults^b^, *N* = 2329		
	n	%		n	%
Age group (in months)			Age group (in years)		
< 12	140	10.7	5–14	1090	46.8
12–23	200	15.3	15–24	385	16.5
24–35	299	22.8	25–34	226	9.7
36–47	313	23.9	35–44	208	8.9
48–59	358	27.3	45–54	160	6.9
			> = 55	260	11.2
Sex			Sex		
Male	696	53.1	Male	859	36.9
Female	614	46.9	Female	1470	63.1
Educational level of respondent^c^			Educational level of respondent^c^		
No education	199	15.2	No education	712	30.6
Primary	494	37.7	Primary	830	35.6
Secondary	542	41.4	Secondary	643	27.6
Tertiary	75	5.7	Tertiary	144	6.2
Occupation of respondent^c^			Occupation of respondent^c^		
Farmer	552	42.1	Farmer	1291	55.4
Trader	498	38.0	Trader	677	29.1
Teacher	45	3.4	Teacher	60	2.6
Civil servant	29	2.2	Civil servant	70	3.0
Others*	186	14.2	Others*	231	9.9
Knowledge/opinion^d^ of respondent^c^			Knowledge/opinion^d^ of respondent^c^		
Poor	117	8.9	Poor	340	14.6
Fair	695	53.1	Fair	1252	53.8
Good	498	38.0	Good	737	31.6

^a^Children and adults (excluding pregnant women) with reported fever/malaria-like illness in the two weeks preceding a survey. ^b^Adults excluding pregnant women. ^c^Respondent female heads of households (the mother of the house or female primary care giver). ^d^Knowledge and opinion level of survey respondents about malaria and malaria diagnosis (knowledge and opinion score of < 50 was poor knowledge, 50–< 70 was fair, > = 70 was good). * Mainly include hair dressers, tailors, house wives, and students

Among the 2329 children ages 5 years and above and adults (excluding pregnant women), the majority of them, 1090 (46.8%), were aged 5–14 years followed by 385 (16.5%) who were aged 15–24 years. The majority of them, 1470 (63.1%) were females. The majority of the respondents, 830 (35.6%), had primary education followed by 712 (30.6%) who had no formal education. Similarly, 1291 (55.4%) were farmers followed by 677 (29.1%) who were traders. Also, 737 (31.6%) had good knowledge and opinion about malaria and malaria diagnosis, 1252 (53.8%) had fair knowledge and opinion, and 340 (14.6%) had poor knowledge and opinion.

### Demand for MRDT by background characteristics

Table 2 presents the demand (use and/or request) for MRDT by background characteristics of participants. Among the overall total of 3639 participants (who had fever/malaria-like illness in the 2 weeks preceding the survey), 1296 (35.6%) received MRDT of which 208 (about 16.1%) requested for the MRDT. Among the 1310 children under 5 years of age, 521 (39.8%) received MRDT of which the caregivers of 82 (15.7%) requested for the MRDT. Among the 2329 children ages 5 years and above and adults (excluding pregnant women), 775 (33.3%) received MRDT of which 126 (16.3%) requested (or their caregivers requested) for the MRDT.

**Table 2:** The demand for MRDT among participants^1^ by background characteristics

	Among participants	Number of participants	Among participants who received MRDT	Number of participants who received MRDT
	% who received MRDT		% who requested** for the MRDT	
Children under 5 years of age				
Age group (in months)				
< 12	35.0	140	4.1	49
12–23	41.5	200	24.1	83
24–35	44.5	299	15.8	133
36–47	37.7	313	14.4	118
48–59	38.5	358	15.9	138
Sex				
Male	42.5	696	17.9	296
Female	36.6	614	12.9	225
Solely visited orthodox providers^5^				
Public sector providers^6^	85.5	433	14.9	370
Private sector providers^7^	10.8	656	18.3	71
Educational level of respondent^3^				
No education	36.7	199	9.6	73
Primary	36.6	494	9.9	181
Secondary	41.7	542	19.5	226
Tertiary	54.7	75	31.7	41
Occupation of respondent^3^				
Farmer	36.6	552	8.4	202
Trader	44.8	498	17.5	223
Teacher	51.1	45	30.4	23
Civil servant	44.8	29	38.5	13
Others*	32.3	186	23.3	60
Knowledge/opinion^4^ of respondent^3^				
Poor	25.6	117	6.7	30
Fair	40.6	695	11.7	282
Good	42.0	498	22.5	209
Total	39.8	1310	15.7	521
Children ages 5 yrs. & above & adults^2^				
Age group (in years)				
5–14	35.7	1090	17.2	389
15–24	31.7	385	18.1	122
25–34	35.4	226	13.7	80
35–44	33.2	208	20.3	69
45–54	26.3	160	11.9	42
> =55	28.1	260	9.6	73
Sex				
Male	33.8	859	16.5	290
Female	33.0	1470	16.1	485
Solely visited orthodox providers^5^				
Public sector providers^6^	88.0	560	17.2	493
Private sector providers^7^	11.6	1130	12.2	131
Educational level of respondent^3^				
No education	26.5	712	9.5	189
Primary	31.1	830	9.7	258
Secondary	38.1	643	22.0	245
Tertiary	57.6	144	34.9	83
Occupation of respondent^3^				
Farmer	28.8	1291	12.1	372
Trader	39.4	677	16.1	267
Teacher	50.0	60	30.0	30
Civil servant	52.9	70	35.1	37
Others*	29.9	231	23.2	69
Knowledge/opinion^4^ of respondent^3^				
Poor	16.2	340	5.5	55
Fair	34.8	1252	15.1	436
Good	38.5	737	20.1	284
Total	33.3	2329	16.3	775
Overall Total	35.6	3639	16.1	1296

^1^Children and adults (excluding pregnant women) with reported fever/malaria-like illness in the two weeks preceding a household survey. ^2^Adults excluding pregnant women. ^3^Respondent female heads of households (the mother of the house or female primary care giver). ^4^Knowledge and opinion level of survey respondents about malaria and malaria diagnosis (knowledge and opinion score of < 50% of the total of 47 was poor knowledge, 50–< 70% was fair, > = 70% was good). * Mainly include hair dressers, tailors, house wives, and students. **Those who requested or whose caregivers requested for MRDT. ^5^Include public and private sector providers (excluding traditional practitioners and drug hawkers). ^6^Include mostly the public primary health centres/providers (others include government hospitals, health posts, community health workers, and free mobile clinics) ^7^ include mostly the patent medicine vendors (others include private hospitals/clinics, private health workers, private mobile clinics, pharmacy)

The proportion of children under 5 years of age who received MRDT was: by far higher among those whose caregivers solely visited public sector providers (370/433, 85.5%) compared with those whose caregivers solely visited private sector providers (71/656, 10.8%); highest among those whose respondents had tertiary education (41/75, 54.7%) and lowest among those with primary education (181/494, 36.6%) and those with no formal education (73/199, 36.7%); and highest among those whose respondents had good knowledge and opinion about malaria and malaria diagnosis (209/498, 42.0%). Similarly, the proportion who requested for the MRDT among those who received MRDT is as shown in Table 2.

The proportion of children ages 5 years and above and adults (excluding pregnant women) who received MRDT was: by far higher among those who (whose caregivers) solely visited public sector providers (493/560, 88.0%) compared with those who (whose caregivers) solely visited private sector providers (131/1130, 11.6%); highest among those whose respondents had tertiary education (83/144, 57.6%) and lowest among those with no formal education (189/712, 26.5%); and highest among those whose respondents had good knowledge and opinion about malaria and malaria diagnosis (284/737, 38.5%). Similarly, the proportion who requested for the MRDT among those who received MRDT is as shown in Table 2.

### Health care-seeking behaviour

**Fig. 1:**
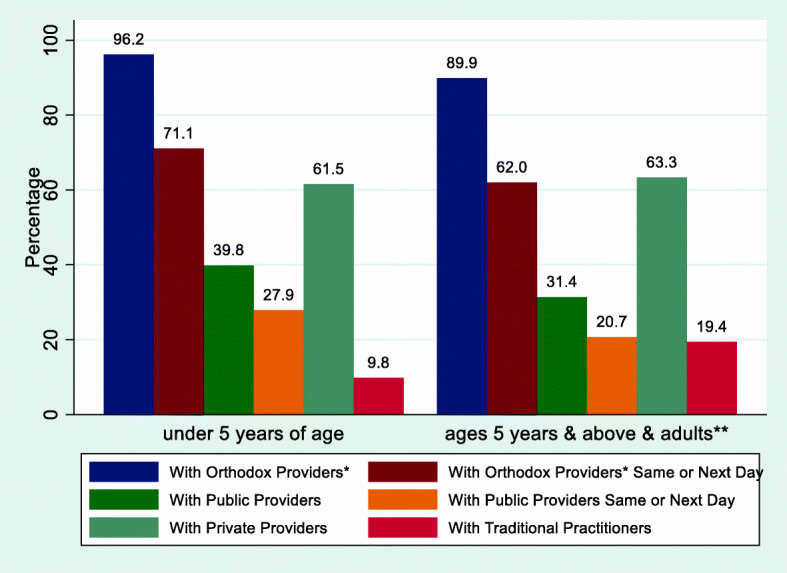
Health care-seeking among participants (children and adults (excluding pregnant women)) with fever/malaria-like illness in the two weeks preceding a survey. *Include public and private sector providers (excluding traditional practitioners and drug hawkers. **Adults excluding pregnant women

The health care-seeking behaviour of participants is presented in Fig. 1. Among the 1310 children under 5 years of age, 1260 (96.2%) sought care with orthodox providers (defined as public and private sector providers excluding traditional practitioners and drug hawkers); 931 (71.1%) sought care with orthodox providers the same or next day; 522 (39.8%) sought care with public sector providers; 365 (27.9%) sought care with public sector providers the same or next day; 806 (61.5%) sought care with private sector providers; and 128 (9.8%) sought care with traditional practitioners. Among the 2329 children ages 5 years and above and adults (excluding pregnant women), 2093 (89.9%) sought care with orthodox providers; 1444 (62.0%) sought care with orthodox providers the same or next day; 731 (31.4%) sought care with public sector providers; 482 (20.7%) sought care with public sector providers the same or next day; 1474 (63.3%) sought care with private sector providers; and 452 (19.4%) sought care with traditional practitioners.

More details of the health care-seeking behaviour of participants are presented in appendix. Among the 1310 children under 5 years of age, 1295 (98.9%) sought care/treatment for the febrile/malaria-like illness. While 495 (37.8%) sought care at government primary health centres, 744 (56.8%) sought care with the PMVs. Those who sought care at more than one places were 169 (12.9%). Among those who sought care at more than one places, 28 (16.6%) first sought care with public sector providers, 91 (53.9%) first sought care with private sector providers, and 50 (29.6%) first sought care with traditional practitioners. While 27 (16.0%) first sought care at government primary health centres, 85 (50.3) first sought care with the PMVs. Among the 2329 children ages 5 years and above and adults (excluding pregnant women), 2268 (97.4%) sought care/treatment for the febrile/malaria-like illness. While 659 (28.3%) sought care at government primary health centre, 1346 (57.8%) sought care with the PMVs. Those who sought care at more than one places were 403 (17.3%). Among those who sought care at more than one places, 74 (18.4%) first sought care with public sector providers, 188 (46.7%) first sought care with private sector providers, and 141 (35.0%) first sought care with traditional practitioners. While 64 (15.9%) first sought care at government primary health centres, 168 (41.7%) first sought care with the PMVs.

### Drug use

**Fig. 2:**
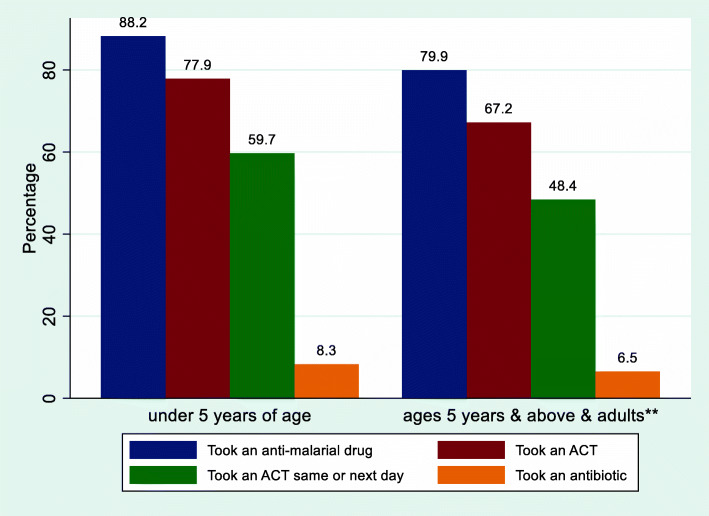
Drug use among participants (children and adults (excluding pregnant women)) with fever/malaria-like illness in the two weeks preceding a survey. **Adults excluding pregnant women

**Fig. 3:**
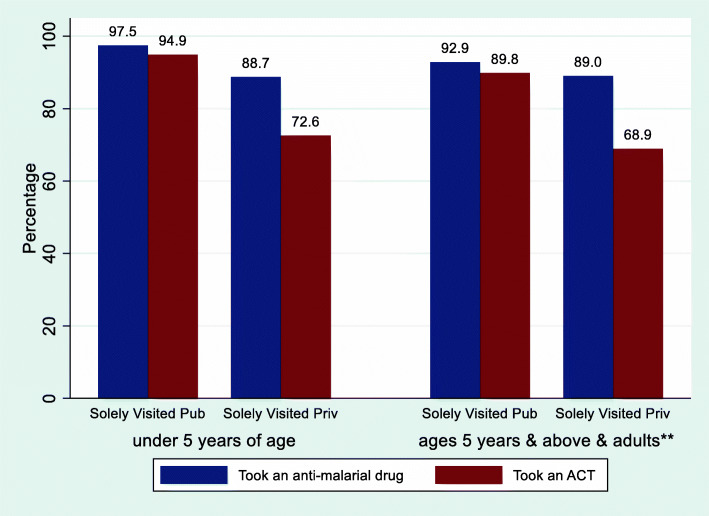
Use of anti-malarial drugs by type of orthodox providers solely visited (orthodox providers were the public and private sector providers excluding traditional practitioners and drug hawkers). Pub = Public providers. Priv = Private providers. **Adults excluding pregnant women

The drug use among the participants is presented in Fig. 2. Among the 1310 children under 5 years of age, 1156 (88.2%) took an anti-malarial drug; 1020 (77.9%) took an ACT (88.2% = 1020/1156 of those who took an anti-malarial drug took an ACT); 782 (59.7%) took an ACT the same or next day; 109 (8.3%) took an antibiotic. Among the 2329 children ages 5 years and above and adults (excluding pregnant women), 1862 (79.9%) took an anti-malarial drug; 1566 (67.2%) took an ACT; 1128 (48.4%) took an ACT the same or next day; 152 (6.5%) took an antibiotic. Use of anti-malarial drugs by type of orthodox providers solely visited by participants is presented in Fig. 3. Among the children under 5 years of age whose caregivers solely visited public sector providers, 97.5% (422/433) took an anti-malarial drug and 94.9% (411/433) took an ACT. Among those whose caregivers solely visited private sector providers, 88.7% (582/656) took an anti-malarial drug and 72.6% (476/656) took an ACT. Among the children ages 5 years and above and adults (excluding pregnant women) who (or whose caregivers) solely visited public sector providers, 92.9% (520/560) took an anti-malarial drug and 89.8% (503/560) took an ACT. Among those who (or whose caregivers) solely visited private sector providers, 89.0% (1006/1130) took an anti-malarial drug and 68.9% (779/1130) took an ACT.

More details of drug use among the participants are presented in appendix. Among the 1310 children under 5 years of age, 1303 (99.5%) took a drug/medicine for the febrile/malaria-like illness; 1150 (87.8%) took paracetamol or ibuprofen; 74 (5.6%) took a traditional medicine (herb or root). Among the 109 that took an antibiotic, 65 (59.6%) took amoxicillin, 20 (18.3%) took co-trimoxazole, 10 (9.2%) took ampicillin, and 6 (5.5%) took ampicillin-cloxacillin. Among the 2329 children ages 5 years and above and adults (excluding pregnant women), 2293 (98.5%) took a drug/medicine for the febrile/malaria-like illness; 1856 (79.7%) took paracetamol or ibuprofen; 329 (14.1%) took a traditional medicine (herb or root). Among the 152 that took an antibiotic, 97 (63.8%) took amoxicillin, 12 (7.9%) took co-trimoxazole, 9 (5.9%) took ampicillin, 8 (5.3%) took ampicillin-cloxacillin, and 11 (7.2%) took ciprofloxacin.

### Factors associated with the demand for MRDT

The associations between the demand for MRDT and the socio-demographic or background characteristics of participants are presented in Table 3. For each factor (characteristic), crude and adjusted odds ratios and their respective 95% CI and *p*-values are reported for each of the other categories compared to a reference category. The crude and adjusted p-values of the overall effect of each factor are also reported.

**Table 3:** Factors associated with the demand for MRDT among participants^1^

Factors	The use of MRDT				The request for MRDT			
	cOR (95% CI)	*p*-value	aOR (95% CI)	*p*-value	cOR (95% CI)	*p*-value	aOR (95% CI)	*p*-value
Children under 5 years of age								
Age group (months)		0.2752^A^		0.1566^A^		0.0783^A^		0.0892^A^
< 12	1.0	–	1.0	–	1.0	–	1.0	–
12–23	1.3 (0.8–2.1	0.227	1.3 (0.8–2.1)	0.230	7.5 (1.7–33.5)	0.009	8.5 (1.8–40.8)	0.007
24–35	1.5 (0.9–2.2)	0.061	1.6 (1.0–2.4)	0.037	4.4 (0.9–19.5)	0.051	6.7 (1.4–32.1)	0.017
36–47	1.1 (0.7–1.7)	0.582	1.1 (0.7–1.7)	0.643	3.9 (0.8–17.8)	0.073	4.8 (1.0–22.5)	0.049
48–59	1.2 (0.8–1.8)	0.463	1.2 (0.8–1.8)	0.419	4.5 (1.0–19.7)	0.049	5.7 (1.2–26.8)	0.027
Sex		–		–		–		–
Male	1.3 (1.0–1.5)	0.030	1.2 (0.9–1.5	0.060	1.5 (0.9–2.4)	0.121	1.4 (0.8–2.4)	0.184
Female	1.0	–	1.0	–	1.0	–	1.0	–
Educ. level of resp^3^		0.0155^A^		0.0550^A^		0.0012^A^		0.2705^A^
No education	1.0	–	1.0	–	1.0	–	1.0	–
Primary	0.9 (0.7–1.4)	0.991	0.9 (0.6–1.3)	0.502	1.04 (0.4–2.6)	0.931	0.8 (0.3–2.1)	0.645
Secondary	1.2 (0.8–1.7)	0.218	1.1 (0.7–1.6)	0.645	2.3 (0.9–5.3)	0.056	1.3 (0.5–3.4)	0.595
Tertiary	2.1 (1.2–3.6)	0.008	2.0 (1.0–4.0)	0.041	4.4 (1.6–12.1)	0.005	2.2 (0.6–8.0)	0.234
Occupation of resp^3^		0.0058^A^		0.0281^A^		0.0011^A^		0.2142^A^
Farmer	1.0	–	1.0	–	1.0	–	1.0	–
Trader	1.4 (1.1–1.8)	0.007	1.3 (0.9–1.7)	0.065	2.3 (1.3–4.2)	0.007	1.9 (0.9–3.8)	0.050
Teacher	1.8 (0.9–3.3)	0.056	1.1 (0.5–2.1)	0.900	4.8 (1.7–13.2)	0.003	1.9 (0.5–6.9)	0.322
Civil servant	1.4 (0.7–2.9)	0.373	0.8 (0.3–1.9)	0.638	6.8 (2.0–23.1)	0.002	3.9 (0.8–17.0)	0.073
Others*	0.8 (0.6–1.2)	0.286	0.7 (0.5–1.1)	0.100	3.3 (1.5–7.2)	0.003	2.3 (0.9–5.4)	0.064
Knowledge/opinion^4^ of respondent^3^		0.0050^A^		0.0254^A^		0.0025^A^		0.0397^A^
Poor	1.0	–	1.0	–	1.0	–	1.0	–
Fair	2.0 (1.3–3.1)	0.002	1.8 (1.2–2.9)	0.008	1.9 (0.4–8.1)	0.413	1.2 (0.3–5.5)	0.822
Good	2.1 (1.3–3.3)	0.001	1.8 (1.2–2.9)	0.011	4.1 (0.9–17.7)	0.062	2.3 (0.5–10.6)	0.292
Children ages 5 yrs. & above & adults^2^								
Age group (years)		0.0643^A^		0.5723^A^		0.4673^A^		0.6065^A^
5–14	1.4 (1.1–1.9)	0.021	1.03 (0.7–1.4)	0.838	2.0 (0.9–4.5)	0.108	1.2 (0.5–3.0)	0.651
15–24	1.2 (0.8–1.7)	0.328	0.9 (0.6–1.4)	0.785	2.1 (0.8–5.1)	0.114	1.4 (0.5–3.6)	0.495
25–34	1.4 (0.9–2.1)	0.084	0.9 (0.6–1.5)	0.913	1.5 (0.5–4.1)	0.427	0.8 (0.3–2.3)	0.679
35–44	1.3 (0.8–1.9)	0.234	0.8 (0.6–1.3)	0.561	2.4 (0.9–6.4)	0.079	1.8 (0.6–4.9)	0.288
45–54	0.9 (0.6–1.4)	0.684	0.7 (0.5–1.1)	0.169	1.3 (0.4–4.3)	0.696	1.03 (0.3–3.7)	0.962
> =55	1.0	–	1.0	–	1.0	–	1.0	–
Sex		–		–		–		–
Male	1.04 (0.8–1.2)	0.705	0.9 (0.8–1.2)	0.953	1.03 (0.7–1.5)	0.864	1.04 (0.7–1.6)	0.868
Female	1.0	–	1.0	–	1.0	–	1.0	–
Educ. level of resp^3^		< 0.0001^A^		0.0001^A^		< 0.0001^A^		0.0001^A^
No education	1.0	–	1.0	–	1.0	–	1.0	–
Primary	1.2 (0.9–1.6)	0.050	1.1 (0.9–1.4)	0.340	1.02 (0.5–1.9)	0.953	0.9 (0.4–1.7)	0.670
Secondary	1.7 (1.4–2.1)	< 0.001	1.4 (1.1–1.8)	0.018	2.7 (1.5–4.8)	0.001	2.3 (1.2–4.5)	0.018
Tertiary	3.8 (2.6–5.5)	< 0.001	2.9 (1.8–4.7)	< 0.001	5.1 (2.6–9.9)	< 0.001	3.9 (1.6–9.3)	0.002
Occupation of resp^3^		< 0.0001^A^		0.0112^A^		0.0006^A^		0.8112^A^
Farmer	1.0	–	1.0	–	1.0	–	1.0	–
Trader	1.6 (1.3–1.9)	< 0.001	1.3 (1.1–1.6)	0.014	1.4 (0.9–2.2)	0.148	1.04 (0.6–1.7)	0.867
Teacher	2.5 (1.5–4.2)	0.001	1.1 (0.6–2.0)	0.751	3.1 (1.3–7.2)	0.008	1.1 (0.4–3.0)	0.816
Civil servant	2.8 (1.7–4.5)	< 0.001	1.2 (0.7–2.2)	0.479	3.9 (1.9–8.3)	< 0.001	1.3 (0.5–3.2)	0.640
Others*	1.1 (0.8–1.4)	0.745	0.8 (0.5–1.1)	0.119	2.2 (1.2–4.2)	0.016	1.5 (0.8–3.1)	0.240
Knowledge/opinion^4^ of resp^3^		< 0.0001^A^		< 0.0001^A^		0.0238^A^		0.1671^A^
Poor	1.0	–	1.0	–	1.0	–	1.0	–
Fair	2.8 (2.0–3.8)	< 0.001	2.5 (1.8–3.4)	< 0.001	3.1 (0.9–10.2)	0.064	3.0 (0.9–10.3)	0.072
Good	3.2 (2.3–4.5)	< 0.001	2.5 (1.8–3.5)	< 0.001	4.4 (1.3–14.4)	0.016	3.3 (0.9–11.2)	0.059

cOR = crude odds ratio from bivariate logistic regression. aOR = adjusted odds ratio from multivariate logistic regression controlling for the other factors (age group, sex, educational level, occupation, and knowledge/opinion level). ^A^*p*-value from overall test for the effect of the independent variables (using the postestimation test command in Stata). * Mainly include hair dressers, tailors, house wives, and students. ^1^Children and adults (excluding pregnant women) with reported fever/malaria-like illness in the two weeks preceding a survey. ^2^Adults excluding pregnant women. ^3^Respondent female heads of households (the mother of the house or female primary care giver). ^4^Knowledge and opinion level of survey respondents about malaria and malaria diagnosis (knowledge and opinion score of < 50 was poor knowledge, 50–< 70 was fair, > = 70 was good)

Among the children under

5 years of age, the factors that were significantly associated with (the predictors of) the use of MRDT were the knowledge and opinion level of respondent female heads of households about malaria and malaria diagnosis (adjusted p-value of overall effect = 0.0254) and occupation of respondents (adjusted p-value of overall effect = 0.0281). The predictor of the request for MRDT was the knowledge and opinion level of respondents (adjusted *p*-value of overall effect = 0.0397). Among the children ages 5 years and above and adults (excluding pregnant women), the predictors of the use of MRDT were the knowledge and opinion level of respondents (adjusted *p*-value of overall effect< 0.0001); educational level of respondents (adjusted p-value of overall effect = 0.0001); and occupation of respondents (adjusted p-value of overall effect = 0.0112). The predictor of the request for MRDT was the educational level of respondents (adjusted *p*-value of overall effect = 0.0001).

### Factors associated with health care-seeking behaviour

The associations between the socio-demographic or background characteristics of participants and health care-seeking with orthodox providers the same or next day and health care-seeking with public providers the same or next day are presented in Table 4. Among the children under 5 years of age, the factors that were significantly associated with (the predictors of) health care-seeking with orthodox providers the same or next day were the knowledge and opinion level of respondent female heads of households about malaria and malaria diagnosis (adjusted *p*-value of overall effect = 0.0064) and occupation of respondents (adjusted p-value of overall effect = 0.0060A). None of the factors assessed was a predictor of health care-seeking with public (orthodox) providers the same or next day.

**Table 4:** Factors associated with health care-seeking behaviour among participants^1^

Factors	Care-seeking with orthodox providers5 the same or next day				Care-seeking with public providers6 the same or next day			
	cOR (95% CI)	p-value	aOR (95% CI)	p-value	cOR (95% CI)	p-value	aOR (95% CI)	p-value
Children under 5 years of age								
Age group (months)		0.7708^A^		0.7453^A^		0.7130^A^		0.6445^A^
< 12	1.0	–	1.0	–	1.0	–	1.0	–
12–23	0.9 (0.5–1.4)	0.603	0.9 (0.6–1.5)	0.716	0.9 (0.6–1.5)	0.782	0.9 (0.6–1.6)	0.847
24–35	0.8 (0.5–1.2)	0.256	0.8 (0.5–1.3)	0.357	1.1 (0.7–1.8)	0.582	1.2 (0.8–1.9)	0.453
36–47	0.9 (0.6–1.4)	0.660	0.9 (0.6–1.5)	0.919	1.1 (0.7–1.6)	0.845	1.03 (0.7–1.6)	0.882
48–59	0.9 (0.6–1.4)	0.735	1.01 (0.6–1.6)	0.986	0.9 (0.6–1.4)	0.623	0.9 (0.6–1.4)	0.680
Sex		–		–		–		–
Male	0.8 (0.7–1.1)	0.156	0.8 (0.6–1.02)	0.072	1.1 (0.9–1.4)	0.319	1.1 (0.9–1.4)	0.460
Female	1.0	–	1.0	–	1.0	–	1.0	–
Educ. level of resp^3^		0.1559^A^		0.7074^A^		0.0175^A^		0.0740^A^
No education	1.0	–	1.0	–	1.0	–	1.0	–
Primary	1.3 (0.9–1.8)	0.207	1.1 (0.7–1.5)	0.726	1.3 (0.9–1.9)	0.233	1.2 (0.8–1.8)	0.344
Secondary	1.5 (1.0–2.1)	0.027	1.1 (0.8–1.7)	0.568	1.3 (0.9–1.9)	0.196	1.3 (0.8–1.9)	0.283
Tertiary	1.5 (0.8–2.6)	0.208	0.8 (0.4–1.6)	0.503	2.5 (1.4–4.4)	0.002	2.6 (1.3–5.2)	0.009
Occupation of resp^3^		0.0017^A^		0.0060^A^		0.0946^A^		0.3074^A^
Farmer	1.0	–	1.0	–	1.0	–	1.0	–
Trader	1.6 (1.2–2.1)	< 0.001	1.6 (1.2–2.1)	0.003	1.2 (0.9–1.6)	0.111	1.1 (0.9–1.5)	0.381
Teacher	2.8 (1.2–6.3)	0.015	3.2 (1.3–8.2)	0.015	1.6 (0.8–3.0)	0.171	0.9 (0.4–2.0)	0.867
Civil servant	1.6 (0.7–3.8)	0.282	1.9 (0.7–5.1)	0.200	1.7 (0.8–3.8)	0.164	0.9 (0.4–2.4)	0.931
Others*	1.1 (0.8–1.6)	0.590	1.1 (0.7–1.6)	0.747	0.8 (0.6–1.2)	0.341	0.7 (0.5–1.1)	0.143
Knowledge/opinion^4^ of respondent^3^		0.0019^A^		0.0064^A^		0.4236^A^		0.8029^A^
Poor	1.0	–	1.0	–	1.0	–	1.0	–
Fair	2.1 (1.4–3.1)	< 0.001	1.9 (1.3–2.9)	0.002	1.3 (0.8–2.0)	0.292	1.2 (0.7–1.9)	0.531
Good	1.8 (1.2–2.7)	0.005	1.6 (1.1–2.5)	0.025	1.4 (0.9–2.2)	0.191	1.2 (0.7–1.9)	0.518
Children ages 5 yrs. & above & adults^2^								
Age group (years)		0.0001^A^		0.0001^A^		0.0003^A^		0.0045^A^
5–14	1.7 (1.3–2.2)	< 0.001	1.5 (1.1–2.0)	0.013	1.9 (1.3–2.7)	0.001	1.4 (0.9–2.1)	0.084
15–24	1.2 (0.9–1.7)	0.195	1.2 (0.8–1.6)	0.359	1.5 (1.0–2.3)	0.048	1.3 (0.8–2.0)	0.237
25–34	1.1 (0.8–1.6)	0.567	0.9 (0.6–1.4)	0.723	1.4 (0.9–2.3)	0.155	1.01 (0.6–1.7)	0.960
35–44	0.9 (0.7–1.4)	0.740	0.8 (0.5–1.1)	0.147	1.1 (0.7–1.9)	0.606	0.8 (0.5–1.4)	0.410
45–54	1.1 (0.7–1.6)	0.765	0.9 (0.6–1.4)	0.772	0.8 (0.5–1.5)	0.542	0.7 (0.4–1.2)	0.190
> =55	1.0	–	1.0	–	1.0	–	1.0	–
Sex								
Male	1.04 (0.9–1.2)	0.696	0.9 (0.7–1.1)	0.214	1.1 (0.9–1.3)	0.580	0.9 (0.7–1.2)	0.493
Female	1.0	–	1.0	–	1.0	–	1.0	–
Educational level of resp^3^		0.0007^A^		0.0041^A^		< 0.0001^A^		< 0.0001^A^
No education	1.0	–	1.0	–	1.0	–	1.0	–
Primary	1.1 (0.9–1.3)	0.495	1.1 (0.8–1.3)	0.671	1.1 (0.9–1.5)	0.350	1.1 (0.8–1.4)	0.648
Secondary	1.4 (1.1–1.8)	0.002	1.5 (1.1–1.9)	0.006	1.7 (1.3–2.2)	< 0.001	1.5 (1.1–2.1)	0.009
Tertiary	1.9 (1.3–2.8)	0.002	2.0 (1.2–3.3)	0.007	3.8 (2.6–5.6)	< 0.001	3.3 (2.0–5.4)	< 0.001
Occupation of resp^3^		< 0.0001^A^		< 0.0001^A^		< 0.0001^A^		0.0032^A^
Farmer	1.0	–	1.0	–	1.0	–	1.0	–
Trader	1.3 (1.1–1.6)	0.005	1.1 (0.9–1.4)	0.362	1.3 (1.1–1.7)	0.010	1.03 (0.8–1.3)	0.802
Teacher	1.4 (0.8–2.4)	0.249	0.8 (0.4–1.5)	0.459	2.3 (1.4–4.1)	0.002	0.9 (0.5–1.7)	0.717
Civil servant	1.6 (0.9–2.7)	0.080	0.9 (0.5–1.8)	0.913	2.9 (1.8–4.8)	< 0.001	1.1 (0.6–2.0)	0.734
Others*	0.6 (0.4–0.8)	< 0.001	0.4 (0.3–0.6)	< 0.001	0.7 (0.5–1.04)	0.080	0.5 (0.3–0.7)	< 0.001
Knowledge/opinion^4^ of resp^3^		< 0.0001^A^		< 0.0001^A^		< 0.0001^A^		< 0.0001^A^
Poor	1.0	–	1.0	–	1.0	–	1.0	–
Fair	2.3 (1.8–2.9)	< 0.001	2.2 (1.7–2.8)	< 0.001	3.4 (2.2–5.2)	< 0.001	3.1 (2.0–4.8)	< 0.001
Good	2.0 (1.6–2.6)	< 0.001	1.8 (1.3–2.3)	< 0.001	4.4 (2.8–6.8)	< 0.001	3.4 (2.1–5.3)	< 0.001

cOR = crude odds ratio from bivariate logistic regression. aOR = adjusted odds ratio from multivariate logistic regression controlling for the other factors (age group, sex, educational level, occupation, and knowledge/opinion level). ^A^p-value from overall test for the effect of the independent variables (using the postestimation test command in Stata). *Mainly include hair dressers, tailors, house wives, and students. ^1^Children and adults (excluding pregnant women) with fever/malaria-like illness in the two weeks preceding a survey. ^2^Adults excluding pregnant women. ^3^Respondent female heads of households (the mother of the house or female primary care giver). ^4^Knowledge and opinion level of survey respondents about malaria and malaria diagnosis (knowledge and opinion score of < 50 was poor knowledge, 50–< 70 was fair, > = 70 was good). ^5^Include public and private sector providers (excluding traditional practitioners and drug hawkers). ^6^Include mostly the public primary health centres/providers (others include government hospitals, health posts, community health workers, and free mobile clinics)

Among the children ages 5 years and above and adults (excluding pregnant women), the predictors of health care-seeking with orthodox providers the same or next day were the knowledge and opinion level of respondents (adjusted p-value of overall effect< 0.0001); educational level of respondents (adjusted p-value of overall effect = 0.0041); occupation of respondents (adjusted p-value of overall effect< 0.0001) and age group of respondents (adjusted p-value of overall effect = 0.0001). The predictors of health care-seeking with public providers the same or next day were the knowledge and opinion level of respondents (adjusted p-value of overall effect< 0.0001), educational level of respondents (adjusted p-value of overall effect< 0.0001), occupation of respondents (adjusted p-value of overall effect = 0.0032), and age group of respondents (adjusted p-value of overall effect = 0.0045).

## Discussion

This study assessed the demand for malaria rapid diagnostic test, health care-seeking behaviour, and drug use among rural community members with reported fever/malaria-like illness, in the 2 weeks preceding a survey, in Ebonyi state, Nigeria. Overall, 35.6% of the community members received MRDT. Particularly, 39.8% of the children under 5 years of age received MRDT while 33.3% of the children ages 5 years and above and adults (excluding pregnant women) received MRDT. However, only 17.9% of the children below 5 years of age in Ebonyi state (and 13.8% nationally) received an MRDT (or a diagnostic test) in a 2018 national survey [12]. This difference could be explained by the fact that, unlike the national survey, our study involved only communities/villages where MRDT services were available because these villages were proximate catchment areas for public primary health facilities and PMVs that had MRDT kits. However, in the national survey clusters were selected without considerations to the availability of MRDT services. In a study in one local government area (LGA) in Kaduna state (Nigeria) [16], 31.0% of the under 5 years old children, who sought care for febrile illness within 48 h, received a diagnostic test. Although this value is just slightly lower than the 39.8% above, it might not be the true picture of the entire Kaduna state since only one LGA was involved in that study.

In this study, even though MRDT services were already available, and free-of-charge at the public primary health centres (due to the efforts of the government and foreign partners), the MRDT test rate was still low (35.6%). This implies widespread presumptive diagnosis and over-diagnosis of malaria as these services were not being optimally utilized. This indicates that more needs to be done to further increase diagnostic testing in addition to the scaling-up of MRDT kits availability and the provision of free MRDT services at public primary health centres.

In this study, 98.9% of the children under 5 years of age sought care/treatment for the febrile/malaria-like illness, 96.2% sought care with orthodox providers, 71.1% sought care with orthodox providers the same or next day, 39.9% sought care with public sector providers, 61.5% sought care with private sector providers, 10.4% sought care with traditional practitioners. While 37.8% sought care at government primary health centres, 56.8% sought care with the PMVs. Contrasting and lower values were reported by a 2018 national survey [12] for the children under 5 years of age in Ebonyi state for all the above health care-seeking behaviours. For example, only 64.3% sought care with orthodox providers and only 36.6% sought care with orthodox providers the same or next day. However, like in our study, the majority sought care with the private sector and with the PMVs. Perhaps the orthodox health facilities or providers were closer to the rural participants in our study compared to the national survey. This might be so because, unlike in the national survey, only clusters that were proximate catchment areas for public primary health facilities and PMVs were used in our study. However, it is not clear whether this could fully explain the higher values in our study because the national survey also involved participants in urban areas where availability and closeness of health facilities would expectedly be relatively high. Also, in a study in one LGA in Kaduna state (Nigeria) [16] only 35.2% of the under 5 years old children sought care for febrile illness within 48 h.

Lower values were also reported by other studies. In a study in North-West Ethiopia [17], 6.8% sought care with traditional healers. The results of a study in Laos [18] show that while 92.0% of heads of households sought treatment for febrile illness, 66.7% sought treatment with orthodox providers. In another study in Ethiopia [19], 76.2% sought care with formal public and private providers. Another study in West Ethiopia [20] reported that 87.8% sought treatment of which only 38.7% did so within 24 h of illness onset. Contrary to the finding in our study, majority in the West Ethiopia study [20] sought treatment with public sector providers.

Although the overall rate of health care-seeking was encouraging in this study, majority sought care (and first sought care) with the PMVs. This would imply a high rate of over-diagnosis and over-treatment of malaria in these communities because studies have reported that over-diagnosis and over-treatment for malaria with ACTs was more among patients visiting the PMVs. This evidence is corroborated by our finding that while only 10.8% of the children under 5 years of age whose caregivers solely visited private sector providers (most of which were PMVs) received MRDT, 72.6% of them took an ACT. This contrasted with the fact that the values for both variables were much closer to each other (85.5% received MRDT and of 94.9% took an ACT) among those whose caregivers solely visited the public sector providers (most of which were government health centres).

In this study, 88.2% of the children under 5 years of age took an anti-malarial drug, 77.9% took an ACT (88.2% of those who took an anti-malarial drug took an ACT), 59.7% took an ACT the same or next day, 87.8% took paracetamol or ibuprofen, 8.3% took an antibiotic, 5.6% took a traditional medicine (herb or root). In a 2018 national survey [12], in the South-East geopolitical zone (which include Ebonyi state), a slightly lower proportion (77.4%) of the children under 5 years of age who took any anti-malarial drug took an ACT. However, similar proportion (7.2%) of these children in Ebonyi state took an antibiotic. Also, in a study in one LGA in Kaduna state (Nigeria) [16] a lower proportion (45.2%) of the under 5 years old children, who sought care for febrile illness within 48 h, received ACT. In a study in Benin [21], a much lower proportion took ACT and paracetamol while a much higher proportion took traditional medicine (herbal tea). In another study in Uganda [22], a lower proportion (26.3%) of those who took an anti-malarial drug took an ACT.

The relatively high rate of ACT use for febrile illness in this study despite the low rate of MRDT (diagnostic testing for malaria) was an indication of over-diagnosis and over-treatment based on presumptive diagnosis. This was not surprising since the majority of the community members sought treatment with the PMVs. For example, among the children under 5 years of age, while only 10.8% of those whose caregivers solely visited private sector providers ((most of which were PMVs) received MRDT, 72.6% of them took an ACT. Even among those whose caregivers solely visited public sector providers, while 85.5% received MRDT, a higher proportion of 94.9% took an ACT. Perhaps this indicated non-adherence to negative MRDT results by many of the public sector providers. Similar picture was also observed among the children ages 5 years and above and adults (excluding pregnant women).

In this study, among the children under 5 years of age, the predictors of health care-seeking with orthodox providers the same or next day were the knowledge and opinion level of respondent female heads of households about malaria and malaria diagnosis and occupation of respondents. Consistent findings were reported by other studies. In a study in West Ethiopia [20] knowledge and perception of malaria were significantly associated with treatment-seeking behaviuor; in another study in northwest Ethiopia [23] knowledge of malaria was significantly associated with early treatment-seeking. The occupation of the respondent female heads of households as a predictor of health care-seeking could be related to occupation as a proxy for socioeconomic status.

The limitation in this study was the measurement of outcomes via the interviewing of respondents about past events and there might be recall bias due to respondents not completely having the memory of these events. However, the bias was minimized by restricting the time period to 2 weeks. In addition, within this prior 2 weeks time period, the most recent events, of the past few days, would be much easier for the respondents to remember.

## Conclusions

There was a low demand (use and/or request) for MRDT among rural community members with reported fever or malaria-like illness in Ebonyi state, Nigeria, despite the availability of free MRDT services. While health care-seeking behavior with orthodox providers was generally encouraging, health care-seeking with the public sector providers, such as government primary health centres, was low as majority sought care in the private sector from the PMVs. Even though majority did not receive any diagnostic test for malaria, most took anti-malaria drugs, and mostly ACTs. The most important overarching predictor identified in this study was the knowledge and opinion level of the female heads of households (or the female primary caregivers) about malaria and malaria diagnosis.

These findings emphasize the need for regular community malaria sensitization interventions or campaigns by the Ebonyi state Malaria Elimination Programme to improve the knowledge and opinion of the female heads of households (or the female primary caregivers) about malaria and malaria diagnosis. There is need for further studies on why the free MRDT services at the government health centres were not being optimally utilized; and interventional studies on how to increase health care-seeking at government health centres, increase MRDT test rate among the PMVs, and generate demand for MRDT in the communities.

## Supplementary Information

**Additional file 1.** .

## Data Availability

The datasets used and/or analysed during the current study are available from the corresponding author on reasonable request.

## Abbreviations

MRDT: Malaria rapid diagnostic test
ACTs: Artemisinin-based combination therapies
WHO: World Health Organization
PMVs: patent medicine vendors
