# Supplementary material for: Demand for malaria rapid diagnostic test, health care-seeking behaviour, and drug use among rural community members with fever or malaria-like illness in Ebonyi state, Nigeria: a cross-sectional household survey
Source: BMC Health Serv Res. 2021 Aug 21;21:857. doi: 10.1186/s12913-021-06865-8 (PMC8380369; doi:10.1186/s12913-021-06865-8)
Supplement: Supplementary file 1 — Additional file 1. . [file 12913_2021_6865_MOESM1_ESM.docx]

| Table 1 Health care-seeking behaviour among participants1 | | | |
| --- | --- | --- | --- |
|  | Children under 5 years of age,  N=1310 | Children ages 5 years and above and adults2,  N=2329 | Total  N=3639 |
|  | n (%) | n (%) | n (%) |
| Sought care/treatment | 1295 (98.9) | 2270 (97.5) | 3565 (98.0) |
| Sought care with orthodox providers5 | 1260 (96.2) | 2093 (89.9) | 3353 (92.1) |
| Sought care with orthodox providers5 the same or next day | 931 (71.1) | 1444 (62.0) | 2375 (65.3) |
| Sought care with public sector providers6 | 522 (39.9) | 731 (31.4) | 1253 (34.4) |
| Government primary health centre | 495 (37.8) | 659 (28.3) | 1154 (31.7) |
| Other public | 29 (2.2) | 77 (3.3) | 106 (2.9) |
| Sought care with public sector providers6 the same or next day | 365 (27.9) | 482 (20.7) | 847 (23.3) |
| Sought care with private sector providers7 | 806 (61.5) | 1474 (63.3) | 2280 (62.6) |
| Patent medicine vendors | 744 (56.8) | 1346 (57.8) | 2090 (57.4) |
| Other private | 75 (5.7) | 142 (6.1) | 217 (6.0) |
| Sought care with traditional practitioners | 128 (9.8) | 452 (19.4) | 580 (15.9) |
|  |  |  |  |
| Among those that sought care at more than one places: | N=169 (12.9) | N=403 (17.3) | N=572 (15.7) |
| First sought care with public sector providers6: | 28 (16.6) | 74 (18.4) | 102 (17.8) |
| First sought care at government primary health centres | 27 (16.0) | 64 (15.9) | 91 (15.9) |
| First sought care with private sector providers7: | 91 (53.9) | 188 (46.7) | 279 (48.8) |
| First sought care with the patent medicine vendors | 85 (50.3) | 168 (41.7) | 253 (44.2) |
| First sought care with traditional practitioners | 50 (29.6) | 141 (35.0) | 191 (33.4) |

^1^Children and adults (excluding pregnant women) with reported fever/malaria-like illness in the two weeks preceding a survey. ^2^Adults excluding pregnant women ^5^Include public and private sector providers (excluding traditional practitioners and drug hawkers). ^6^Include mostly the public primary health centres/providers (others include government hospitals, health posts, community health workers, and free mobile clinics) ^7^ include mostly the patent medicine vendors (others include private hospitals/clinics, private health workers, private mobile clinics, pharmacy).

| Table 2 Drug use among participants1 | | | |
| --- | --- | --- | --- |
|  | Children under 5 years of age  N=1310 | Children ages 5 years and above and adults2  N=2329 | Total  N=3639 |
|  | n (%) | n (%) | n (%) |
| Took a drug/medicine | 1303 (99.5) | 2293 (98.5) | 3596 (98.8) |
| Took an anti-malarial drug | 1156 (88.2) | 1862 (79.9) | 3018 (82.9) |
| Toot an ACT | 1020 (77.9) | 1566 (67.2) | 2586 (71.1) |
| Took an ACT the same or next day | 782 (59.7) | 1128 (48.4) | 1910 (52.5) |
| Took paracetamol (or ibuprofen) | 1150 (87.8) | 1856 (79.7) | 3006 (82.6) |
| Took an antibiotic | 109 (8.3) | 152 (6.5) | 261 (7.2) |
| Took a traditional medicine (herb or root) | 74 (5.6) | 329 (14.1) | 403 (11.1) |
| Did not know the medicine that was taken | 11 (0.8) | 54 (2.3) | 65 (1.8) |
|  |  |  |  |
| Among those that took antibiotics: | N=109 | N=152 | N=261 |
| Took amoxicillin | 65 (59.6) | 97 (63.8) | 162 (62.1) |
| Took co-trimoxazole | 20 (18.3) | 12 (7.9) | 32 (12.3) |
| Took ampicillin | 10 (9.2) | 9 (5.9) | 19 (7.3) |
| Took ampicillin-cloxacillin | 6 (5.5) | 8 (5.3) | 14 (5.4) |
| Took ciprofloxacin | 1 (0.9) | 11 (7.2) | 12 (4.6) |
| Took metronidazole | 3 (2.8) | 7 (4.6) | 10 (3.8) |
|  | | | |
| Use of anti-malarial drugs by type of orthodox providers5 solely visited | | | |
|  | Took an anti-malarial drug n/N (%) | | |
| Solely visited public sector providers6 | 422/433 (97.5) | 520/560 (92.9) | 942/993 (94.9) |
| Solely visited private sector providers7 | 582/656 (88.7) | 1006/1130 (89.0) | 1588/1786 (88.9) |
|  | Took an ACT n/N (%) | | |
| Solely visited public sector providers6 | 411/433 (94.9) | 503/560 (89.8) | 914/993 (92.0) |
| Solely visited private sector providers7 | 476/656 (72.6) | 779/1130 (68.9) | 1255/1786 (70.3) |
|  |  |  |  |

^1^Children and adults (excluding pregnant women) with reported fever/malaria-like illness in the two weeks preceding a survey. ^2^Adults excluding pregnant women. ACT=Artemisinin-based combination therapy. ^5^Include public and private sector providers (excluding traditional practitioners and drug hawkers). ^6^Include mostly the public primary health centres/providers (others include government hospitals, health posts, community health workers, and free mobile clinics) ^7^ include mostly the patent medicine vendors (PMVs) (others include private hospitals/clinics, private health workers, private mobile clinics, pharmacy).
